# Type R1 Single Coronary Artery Without Obstructive Disease: A Multimodal Imaging Approach

**DOI:** 10.1016/j.jaccas.2025.104916

**Published:** 2025-08-06

**Authors:** Meena Farid, Inderbir Padda, Arun Mahtani, Daniel Fabian, Charles Sineri

**Affiliations:** a Department of Cardiology, Brookdale University Hospital and Medical Center, Brooklyn, New York, USA; b Department of Internal Medicine, Richmond University Medical Center/Mount Sinai, Staten Island, New York, USA; c Department of Cardiology, Virginia Commonwealth University, Richmond, Virginia, USA; d Department of Cardiology, University of Central Florida/HCA Florida Healthcare, Ocala, Florida, USA; e Department of Cardiology, Richmond University Medical Center/Mount Sinai, Staten Island, New York, USA

**Keywords:** coronary angiography, coronary calcium score, coronary vessel anomaly

## Abstract

Single coronary artery is a congenital anomaly defined by a solitary artery supplying the heart. It may present as angina in the absence of coronary obstruction. A 73-year-old man presented to our institution with chest pain and exertional dyspnea. He was ultimately found to have a single coronary artery arising from the right coronary cusp (type R1). Multimodal imaging, including computed tomography and coronary angiography, revealed no flow-limiting lesions. The patient was medically managed to optimize coronary disease risk and to minimize symptoms. This case underscores the clinical relevance of anatomical coronary variants and the role of imaging in evaluating symptoms in this subset of the population.

## Graphical Abstract

**Figure:**
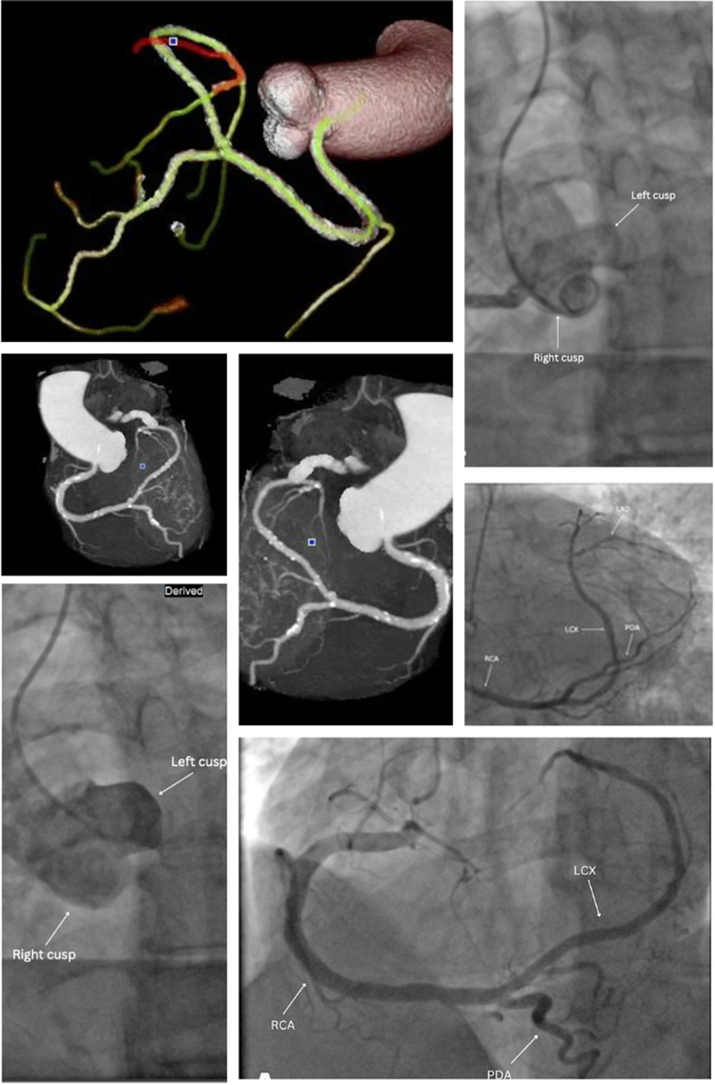

## Case Summary

**Figure 1:**
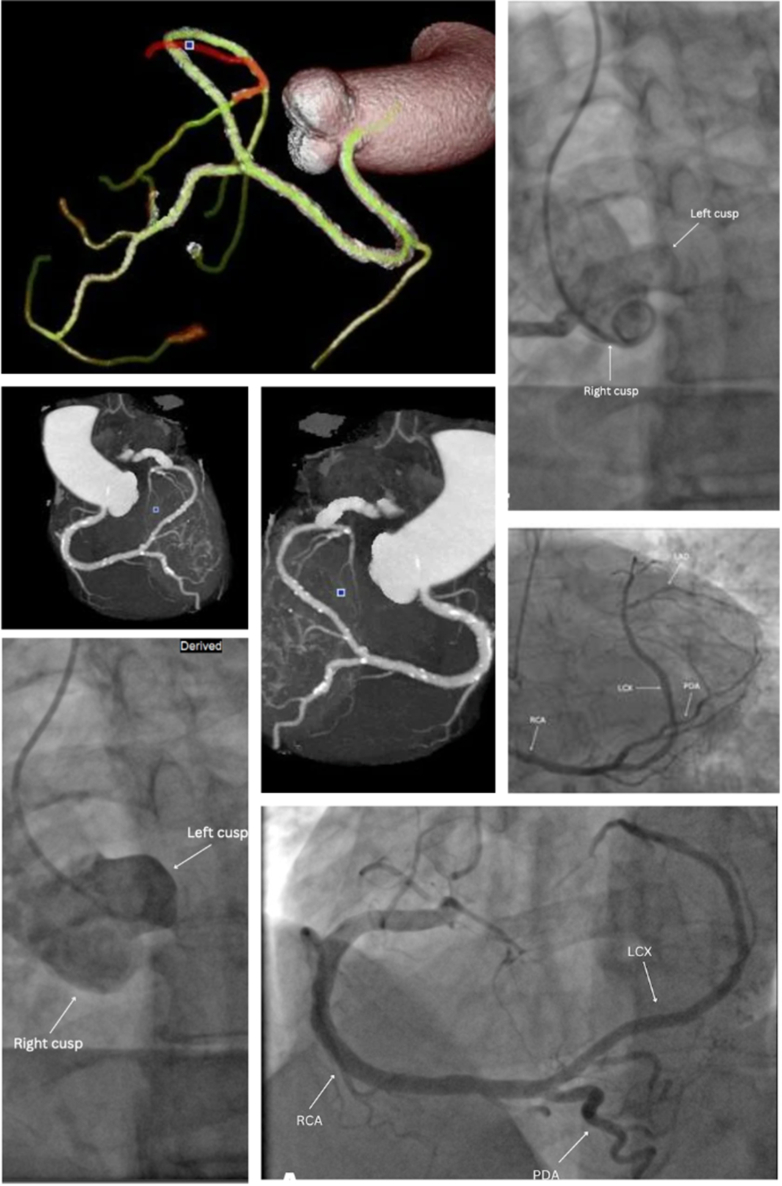
Single Coronary Artery (Type R1) Identified via Computed Tomography Angiography and Coronary Angiography This is a right-dominant coronary arterial system; there is no left main coronary artery. The left anterior descending coronary artery (LAD) and left circumflex coronary artery (LCx) arise from the right coronary artery (RCA). The RCA has proximal scattered calcified and noncalcified plaque causing minimal stenosis. There are also scattered distal calcified and noncalcified plaques causing minimal stenosis. The posterior descending artery (PDA) has scattered calcified plaque. The midsegment of the PDA has a focal calcified plaque causing mild stenosis. The PDA wraps around the apex and is without significant stenosis. The LCx arises as an extension of the RCA. The LCx shows diffusely scattered calcified plaque causing minimal stenosis. This vessel continues to form the LAD. The LAD territory is supplied by the continuation of the RCA from the LCx. The LAD gives rise to the first diagonal branch (D1) and a small-caliber second diagonal branch (D2). At the bifurcation, there is a focal calcified plaque causing minimal stenosis. The LAD is without significant stenosis. There is a proximal D1 calcified plaque limiting luminal analysis and potentially causing moderate stenosis. There is a small-caliber D2 without significant stenosis. A wraparound PDA supplies the apex. Coronary angiograms showing (Top Right) nonselective injection into the right coronary cusp; (Middle Right) right anterior oblique cranial; (Bottom Left) nonselective injection into left coronary cusp; and (Bottom Right) left anterior oblique cranial.

A 73-year-old man with hyperlipidemia and a remote history of tobacco use presented with exertional chest pain and dyspnea. Pain was located in the retrosternal and interscapular area, with nocturnal episodes that were worse with exertion. On examination, he had elevated blood pressure (172/80 mm Hg) and bradycardia (52 beats/min). Electrocardiogram and troponin levels were normal. A regadenoson stress test showed mild reversible ischemia in the distal anterior wall. Transthoracic echocardiography demonstrated a preserved ejection fraction without wall motion abnormalities. Invasive coronary angiography revealed a single coronary artery (SCA) arising from the right coronary cusp without flow-limiting lesion, consistent with a Lipton-Yamanaka type R1 classification (Figure 1). The right coronary artery supplied the entire myocardium, including the territories typically perfused by the left anterior descending artery and left circumflex artery (Figure 1). This R1 type of SCA gave rise to a wraparound posterior descending artery, which supplied the apex (Figure 1). Computed tomography (CT) coronary angiography confirmed this anatomy and revealed mild nonobstructive coronary artery disease (Coronary Artery Disease–Reporting and Data System [CAD-RADS] score of 3, indicating 50%-69% stenosis), with a high calcium score (Agatston score: 675) (Figure 1, Video 1, Video 2, Video 3) The left anterior descending artery was a small-caliber, patent vessel. No pericardial effusion or structural abnormalities were noted. No intervention was indicated owing to absence of significant flow-limiting lesions. The patient was treated with aspirin, a statin, a beta-blocker, and antihypertensives. Close outpatient follow-up was arranged for risk-factor modification and longitudinal assessment.

**Take-Home Messages**

• SCA is a rare congenital anomaly that may present with angina even in the absence of obstructive coronary disease.
• A multimodal imaging approach, including stress testing, coronary angiography, and CT angiography, helps clarify anatomy and guide management.

SCA is an uncommon anomaly (prevalence: 0.024%-0.066%) in which a single ostium gives rise to a coronary artery that perfuses the entire heart. Type R1, with a benign course originating from the right coronary cusp, accounts for approximately 15% of SCA cases.^1^ Although considered less malignant than interarterial courses, R1 variants may still be symptomatic owing to ischemia from increased myocardial demand or atherosclerotic burden.^1^ In our patient, symptoms likely occurred from increased-demand ischemia in the setting of calcific plaque and hypertension. Definitive diagnosis of SCA requires careful imaging interpretation. CT angiography was essential for evaluating the vessel trajectory and excluding a malignant course, which would have required surgical consultation. Whereas invasive angiography delineated luminal anatomy and ruled out significantly obstructive lesions, CT provided further three-dimensional context, identifying vessel paths and structural relationships. Recognition of these anomalies is critical, as revascularization carries elevated procedural risk when the entire myocardium is dependent on a single vessel. Even in the absence of obstructive disease, the unique anatomy necessitates individualized management and long-term follow-up.

## Funding Support and Author Disclosures

The authors have reported that they have no relationships relevant to the contents of this paper to disclose.

## Appendix

Video 1 CT Angiogram Showing a Right-Dominant Coronary Arterial System This is a right-dominant coronary arterial system; there is no left main coronary artery. The left anterior descending coronary artery (LAD) and left circumflex coronary artery (LCx) arise from the right coronary artery (RCA). The RCA has proximal scattered calcified and noncalcified plaque causing minimal stenosis. There are also scattered distal calcified and noncalcified plaques causing minimal stenosis. The posterior descending artery (PDA) has scattered calcified plaque. The midsegment of the PDA has a focal calcified plaque causing mild stenosis. The PDA wraps around the apex and is without significant stenosis. The LCx arises as an extension of the RCA. The LCx shows diffusely scattered calcified plaque causing minimal stenosis. This vessel continues to form the LAD. The LAD territory is supplied by the continuation of the RCA from the LCx. The LAD gives rise to the first diagonal branch (D1) and a small-caliber second diagonal branch (D2). At the bifurcation, there is a focal calcified plaque causing minimal stenosis. The LAD is without significant stenosis. There is a proximal D1 calcified plaque limiting luminal analysis and potentially causing moderate stenosis. There is a small-caliber D2 without significant stenosis. A wraparound PDA supplies the apex. Coronary angiograms showing (top right) nonselective injection into the right coronary cusp; (middle right) right anterior oblique cranial; (bottom left) nonselective injection into left coronary cusp; and (bottom right) left anterior oblique cranial.
